# Artificial intelligence, machine learning, and deep learning for clinical outcome prediction

**DOI:** 10.1042/ETLS20210246

**Published:** 2021-12-20

**Authors:** Rowland W. Pettit, Robert Fullem, Chao Cheng, Christopher I. Amos

**Affiliations:** 1 Institute for Clinical and Translational Research, Baylor College of Medicine, Houston, TX, U.S.A.; 2 Department of Molecular and Human Genetics, Baylor College of Medicine, Houston, TX, U.S.A.; 3 Section of Epidemiology and Population Sciences, Department of Medicine, Baylor College of Medicine, Houston, TX, U.S.A.; 4 Dan L Duncan Comprehensive Cancer Center, Baylor College of Medicine, Houston, TX, U.S.A.

**Keywords:** artificial intelligence, deep learning, machine learning, review

## Abstract

AI is a broad concept, grouping initiatives that use a computer to perform tasks that would usually require a human to complete. AI methods are well suited to predict clinical outcomes. In practice, AI methods can be thought of as functions that learn the outcomes accompanying standardized input data to produce accurate outcome predictions when trialed with new data. Current methods for cleaning, creating, accessing, extracting, augmenting, and representing data for training AI clinical prediction models are well defined. The use of AI to predict clinical outcomes is a dynamic and rapidly evolving arena, with new methods and applications emerging. Extraction or accession of electronic health care records and combining these with patient genetic data is an area of present attention, with tremendous potential for future growth. Machine learning approaches, including decision tree methods of Random Forest and XGBoost, and deep learning techniques including deep multi-layer and recurrent neural networks, afford unique capabilities to accurately create predictions from high dimensional, multimodal data. Furthermore, AI methods are increasing our ability to accurately predict clinical outcomes that previously were difficult to model, including time-dependent and multi-class outcomes. Barriers to robust AI-based clinical outcome model deployment include changing AI product development interfaces, the specificity of regulation requirements, and limitations in ensuring model interpretability, generalizability, and adaptability over time.

## Introduction

**Figure 1.**
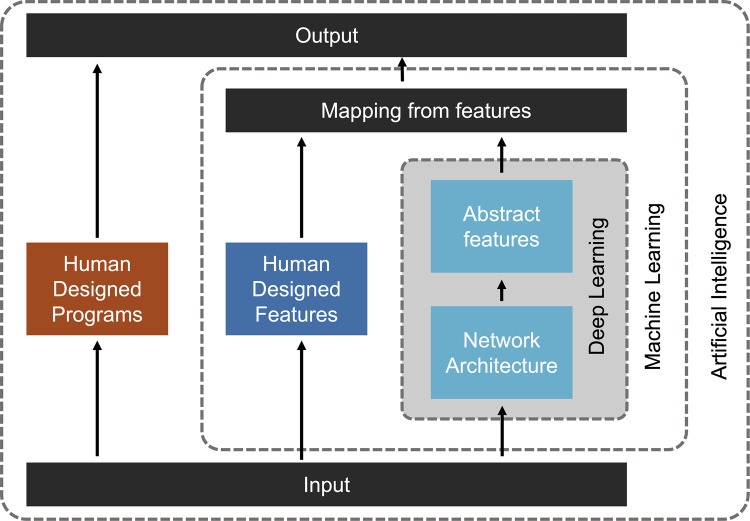
Representation of concepts: artificial intelligence, machine learning, and deep learning.

In the modern era, the volume and variability of data available to understand and predict clinical outcomes are beyond the scope of singular human comprehension. For this reason, artificial intelligence (AI) methods are well-positioned to meaningfully assist in the clinical practice of medicine. AI is a broad concept, grouping together many initiatives that use a computer to perform tasks that would usually require a human to complete [1]. Examples of computational solutions which fall under the category of AI include perceiving visual stimuli, understanding speech, making decisions based on input data, and language translation [2]. ML is a sub-concept of AI, which focuses on having a machine perform an otherwise intelligent task by learning based on its errors to improve its capabilities with experience [3]. ML adapts and learns iteratively, without human feedback, by applying statistical models that identify patterns in data and draw useful inferences [4]. Finally, deep learning (DL) is a specific category within ML that uses various artificial neural network architectures to extract and process features within data. This hierarchy, narrowing in from broad to specific, can be appreciated in Figure 1, adapted from Min et al. [5]. The tangible products in the field of AI have evolved greatly over the last 30 years. We point the reader here for historical [6,7,8,9], technical [10], medically focused [11], and failure highlighting [12], reviews on the evolution of AI methods. This review will outline the progress, uses, and barriers to comprehensively integrating these emerging statistical and machine learning (ML) tools into clinical practice.

## Clinical outcome predictions

### Conceptual framework

Clinical outcome predictive models can be generalized down to the following concept. A model, represented as a function ‘*f’* is applied to input data X to represent a known outcome variable y [13]

f(X)→y

The model *f* is ‘fit,’ or trained on the input data X so that when it encounters new data it can predict what y will be, or ŷ [13]. The goal of creating a clinical outcome predictor is for your *f* to work well enough so that predicted values of ŷ on new data would be correct if the actual status or value of y was known. Input data X can take many forms. However, usually input information is cleaned and processed into matrix form [14]. In this data object, each row, or ‘instance,’ represents a single entity or observation of the data (i.e. an individual patient), and each column, or ‘feature,’ represents a property of the data (i.e. the patient's age, or blood type). Models where only linear operations (*f)* act on input features (X) to predict an outcome (*y)* are referred to as generalized linear models, or GLMs [15]. Various non-linear, or other operations, however, may be implemented on these data (X). In general, a weight, *w,* is given to each feature as the features are combined. Using matrix notation, our general equation can be conceptualized as follows [13]:

f(w⋅X)→y

While the exact weighting and feature manipulation methods (*f)* are unique per ML method, generally, models are built through iterative training [13]. The ML method *f* is initiated with random parameter weights *w_0_,* the model is fit to the input *f(w_0_* **·** *X)*, and an initial outcome prediction ŷ*_0_* is generated. Then a loss function is created, which can take various forms (i.e. root mean squared error [16] or cross-entropy loss [17]) to compare each ŷ prediction to the known outcome *y.* Loss functions (*L)* operate such that the closer the ŷ prediction comes to accurately getting the real value of y, the smaller the function output is [18]. It is then relatively straightforward to optimize an algorithm by minimizing your defined loss function. During iterative training, the gradient of the loss function dictates how the weights of features (w) should be changed so that the loss function is minimized. The goal of loss optimization is for the models’ predictions (ŷ) to get closer and closer to their actual outcome value (y), which is observed as loss function converges to 0 or is minimized:

$$L(y,\hat{y})|w\to 0,\;\min$$

### What is an outcome, and how will you measure it?

With a general framework established, it is next necessary to consider the outcome, y, and decide how it should appropriately be measured. This step is essential as different ML methods lend themselves most appropriately to modeling differing outcome types. For example, the most straightforward clinical outcome that can be observed is that of a binary outcome. A binary outcome variable can only take two values and often represents a ‘yes’/’no’ event [19]. Clinically, these could include an outcome describing treatment failure versus success or patient mortality within a defined time period from an intervention. Almost all ML methods can be used to perform binary classification [13] However, perhaps you are interested in an outcome with more than two classes and need to create a multi-class classifier [20,21]. This could be the case when trying to differentiate among multiple types of dermatological lesions, including benign, melanoma, basal cell, and squamous cell carcinoma lesions. The next step up from a multi-class classifier is creating an ML model that can predict a continuous [22] clinical outcome. Perhaps you wish to predict the expected level for a biomarker or hope to predict the length of stay for patients who receive a procedure. An important consideration for a classifier is understanding if an outcome occurs over a time horizon. Is there a time component to incorporate into the model? For example, when looking at cancer recurrence after chemotherapy, recurrence rates are only an interesting if you know the period of remission, after chemotherapy but before recurrence. ML models can perform ‘survival analysis’ [23] tasks. Utilizing a time-dependent outcome variable comes with constraints, however. Instances where patients are in your data but have not yet experienced an outcome must be ‘censored’ appropriately. Censoring is accomplished in a nonparametric manor in classical statistics through the Kaplan–Meier [24] method. This compensatory method is imperfect, however, and fails to appropriately account for the informative censoring of competing risks [25], where patient dropout may be non-random and censored individuals have risk factors influencing the survival outcome of interest. Other survival analysis specific analyses requirements include assessing the informative missingness [26] of covariates [27], the impact of confounders [28], and latent heterogeneity of patient cohorts [29]. These considerations can be handled to a degree with advanced statistical methods, but not yet by ML methods. Further detailed insights into survival analysis are available in the literature [30,31].

**Figure 2.**
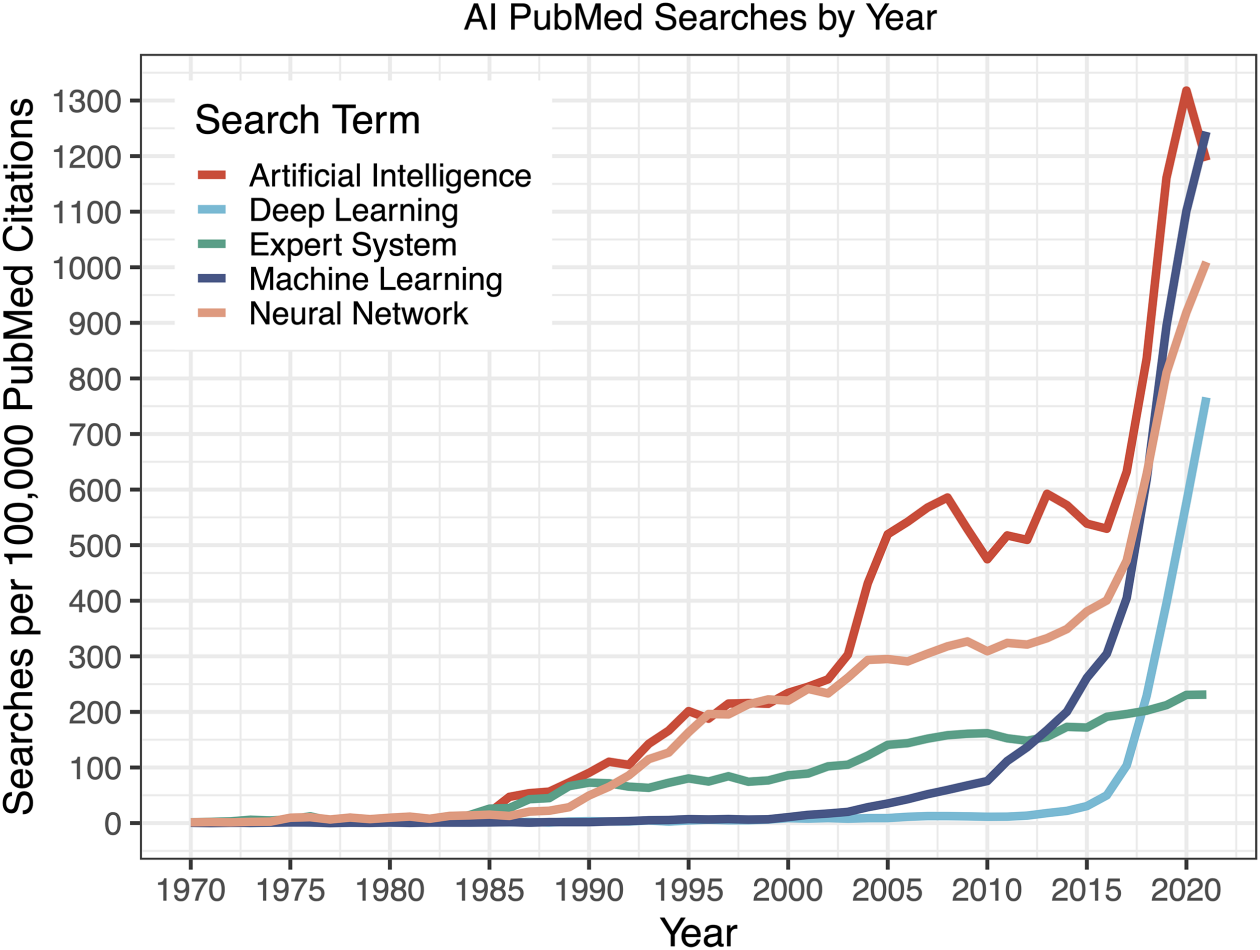
AI PubMed searches per 100 000 citations by Year.

**Figure 3.**
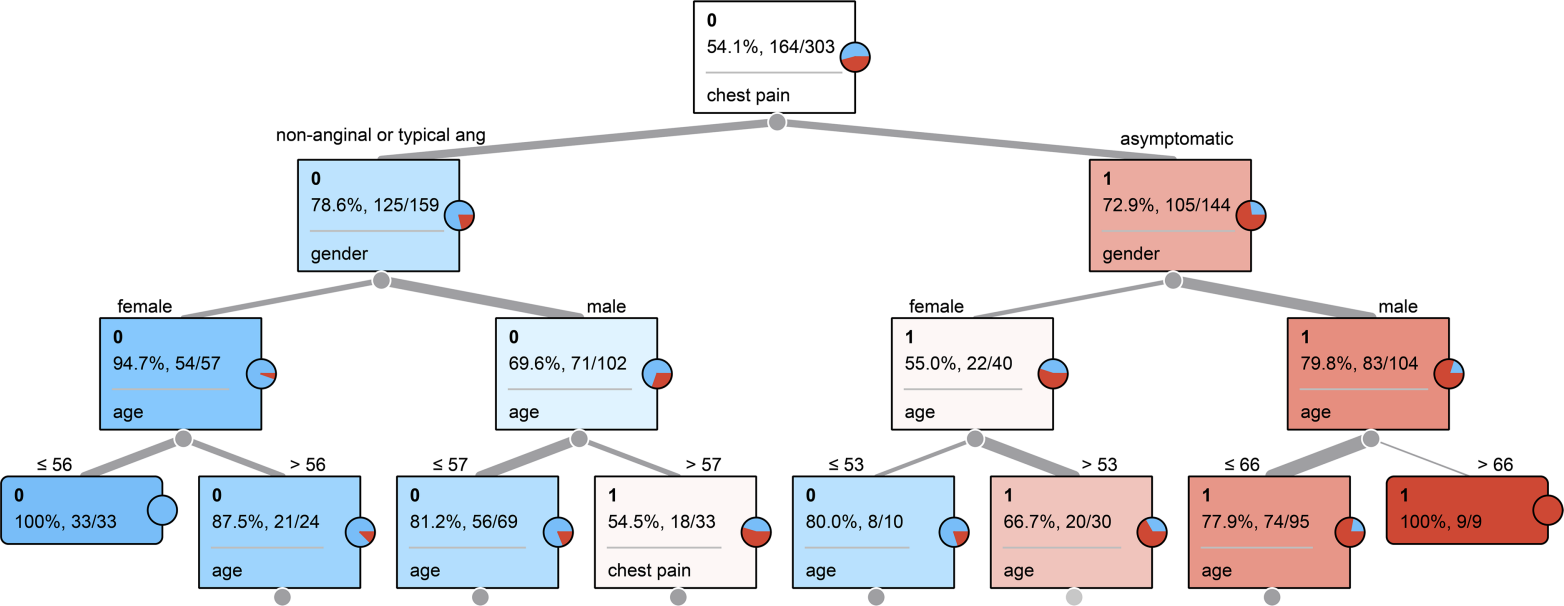
Example of a decision tree to predict coronary artery narrowing (1, red) vs no narrowing (0, blue) using input features of age, gender, and type of chest pain.

## Emerging methods and emerging applications

ML methods are rapidly being applied in novel avenues to predict clinical outcomes. To visualize, we have generated Figure 2 charting AI related PubMed searches by year. In Supplementary Table S1, we have complied recent review articles detailing emerging examples of how statistical and ML methods are being utilized for clinical outcome prediction in major medical specialities. Applications are found in the fields of Anesthesiology [32,33,34], Dermatology [35,36,37], Emergency Medicine [38,39], Family Medicine [40,40], Internal Medicine [41,42,43], Interventional Radiology [44,45], Medical Genetics [46], Neurological Surgery [47], Neurology [48,49,50], Obstetrics and Gynecology [51,52], Ophthalmology [53,54,55], Orthopaedic Surgery [56], Otorhinolaryngology [57,58], Pathology [59,60,61], Pediatrics [62], Physical Medicine and Rehabilitation [63,64], Plastic and Reconstructive Surgery [65,66], Psychiatry [67,68], Radiation Oncology [69,70], Radiology [71,72], General Surgery [73,74], Cardiothoracic Surgery [75,76], Urology [77,78], Vascular Surgery [79,80]. These papers introduce terms describing ML models as ‘supervised’ or ‘unsupervised’. Supervised ML methods are trained to predict a specified outcome, while unsupervised models are given no outcome target and seek to identify patterns in data unguided [81]. Almost all clinical outcome statistical and ML models are supervised models [82] where a target is set beforehand. Let's investigate these methods mentioned individually.

1. *Linear Models.* Logistic and linear regression (LR) are the most straightforward predictive models. These are classical statistical techniques and are most accurately regarded as statistical learning methods [13]. LR methods combine input features in a linear combination to predict an outcome. When input features are independently correlated with the outcome, linear models perform very well, on par or even better than new ML methods [83,84]. LR methods do not capture non-linear relationships between variables, and, without specific feature construction, treat all features independently [85]. LR models will continue to provide excellent insight into clinical outcome predictions as LR models are both computationally efficient and highly interpretable [15]. LR feature weights can be tested for individual significance and be understood as feature multipliers in relation to an outcome metric [86]. It is standard practice to benchmark performance of ML models to that of statistical learning LR methods to critically evaluate the need for a more complex, often less interpretable ML model [87]. As example, LR methods are recently being used to predict clinical outcomes of COVID-19 mortality [88], the development of chronic diseases such as HTN and DM [84], stroke risk [89], and predicting acute myeloid leukemia outcomes from patient gene signatures [90]. When outcomes evolve over time, linear cox-proportional hazards statistical models are used to estimate baseline and feature specific hazard ratios of an outcome continuously [91]. Cox models are statistically entrenched due to interpretability, simplicity, and enduring widespread incorporation [92]. Although not a regression technique, the Naïve Bayes (NB) ML method also appreciates data features independently toward an outcome of interest [93]. For binary outcome prediction, NB calculates the posterior probability of the positive outcome class for each numerical feature and each sub-category within categorical features totaling probabilities [94]. NB has been recently used to predict responses to chemotherapy [95] and to predict the development of Alzheimer's disease from genomic data [96].
2. *Decision Tree Methods.* An individual decision tree is a top-down flowchart-like structure in which nodes represent a decision point, determined by a single input feature, and branches from nodes continue to diverge reaching more terminal nodes (Figure 3). Node decision points are created from features through information theory in which features are split based on entropy or variance regarding the known outcome of interest. After training the outcomes into a decision tree, then a new data instance of information can be fed into the tree, and the node decisions can be followed to predict the likely clinical outcome.

The method may be used to create both Classification and Regression Trees, which produces the acronym CART [97] for brevity. Many tree-based methods exist. A random forest trains multiple decision trees on input data, with each subtree having only a subset of the total column feature variables to consider. After training, all trees of the forest are run in parallel on new data entries, and the majority prediction opinion of the forest determines the model's final prediction. This method has the advantage of making decision nodes to be created at minor features, forcing their appreciation, and avoiding a few strong predictors driving prediction in all scenarios. This ability has led to excellent clinical outcome predictions, including recently to predict stroke outcomes [98], drug response from clinical and serological markers [99], or mortality after traumatic brain injury [100]. On small datasets with only a few highly correlated features, a random forest model may not perform better than simpler methods [101]. Two key concepts are introduced with the random forest. Combining several individual models to create one is known as ensemble modeling. Training multiple base models in parallel is known as ‘bootstrap aggregation,’ or ‘bagging’ [102]. Bagging is used in various statistical applications and does not require decision trees to exclusively serve as the underlying base models [103]. Boosting ensemble methods [61], by contrast, take a different strategy and train multiple models in series. By training sequentially, boosting affords later models the opportunity to learn from the previous models, or ‘learners,’ weaknesses. Popular boosting methods used in clinical modeling include XGBoost [104,105] and AdaBoost [106,107], which are often multi-decision tree ensemble methods [108,109]. Finally, when a time horizon outcome variable is used, novel random survival forest [110] methods can be used for time-dependent clinical predictions [111]. In general, tree-based methods are interpretable, can appropriately model non-linear relationships, and feature rankings of relative importance can be readily retrieved [112]. Tree-based methods are limited in that they require manual feature construction to appreciate multiple variables concurrently [113].

3. *Clustering, Kernel and Non-deterministic* methods. Clustering methods, in general, are unsupervised.

ML methods, however, they can be used for clinical outcome predictions. In the k-Nearest Neighbor (kNN) approach, clusters are found within data through ‘k’ number of random centroid placements, iterative Euclidian distance calculation between all data and centroids, recentering centroids to be in the center of ‘nearest points’, and reassignment of data cluster labels. KNNs have been utilized to cluster large-scale microRNA expression profiles into correctly classified human cancers [114,115,116]. Support vector machines [117] (SVM) are a kernel-based ML method that attempts to represent data into a higher dimensional feature space and find a hyperplane to separate samples by their outcome status [13]. SVM has limited utility when your input data has a large number of dimensions, as projecting all features into a higher dimensional space is computationally intensive, especially when using a non-linear kernels. They are used to predict outcomes when datasets are manageable [118]. Non-Deterministic methods are machine learning methods where a model is not constrained to create predictions in the context of the known outcome. For example, a non-deterministic classifier [119] trained to predict a binary outcome may be allowed to predict three or more states. The advantage of this is that the model has more ‘options’ to bucket borderline negative instances into when it is unsure of the appropriate class designation. This principle can ultimately lead to more correct classifications of the positive class. Such methods have been applied to clinical outcome prediction [120], where they demonstrated utility in predicting clinical cancer type [119]. More information on non-deterministic algorithms may be found here [121].

**Figure 4.**
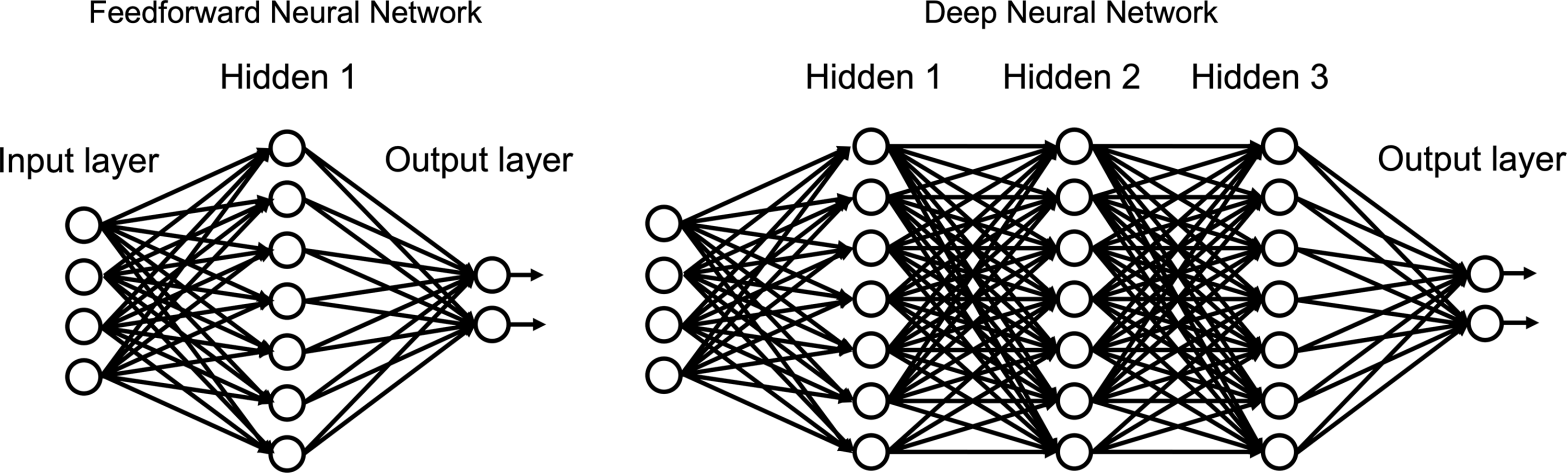
Fully connected (Dense) neural network versus deep neural network.

**Figure 5.**
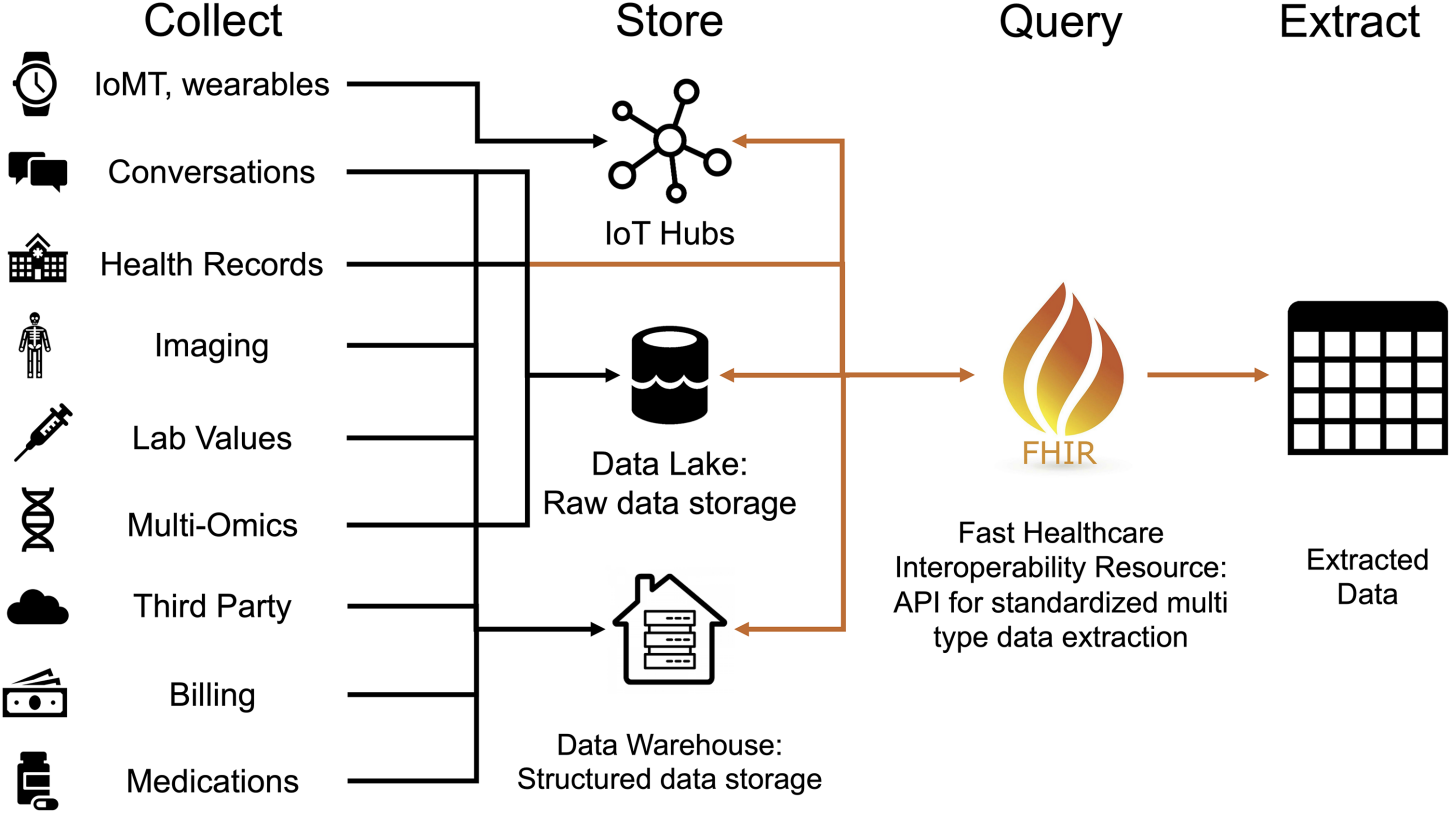
Healthcare data extraction standard pipeline.

4. *Deep Learning.* Deep learning is a sub-category within ML, defined by the use of neural network architectures [5,122]. The most basic neural network architecture is a fully connected, or ‘dense’ [123], feed-forward network, or a ‘multi-layer perceptron’ [125]. In Figure 5 we can see how a deep neural network [124] simply refers to having more than one hidden layer of interconnected nodes. In a general neural network architecture, the value of circles, or nodes, is the weighted sum of the outputs of nodes connected to it [125]. Line connections each have a weight, which is an individual parameter that is tuned during model training, modulated by optimizing your determined loss function [126]. To introduce non-linearity into the network, an activation function (ReLu [127], Sigmoid [128]) acts on threshold weighted inputs into a node. Feedforward neural networks are useful for clinical outcome prediction [129]. Deep neural networks (Figure 4) increase the number of hidden layers [130] between input and output and add advantages of more abstract feature representations [5]. Deep learning methods are being used extensively to predict clinical outcomes [131,132,133] When limited training instances are available, transfer learning [134] is appropriate. In transfer learning, a deep neural network model is pre-trained on a large adjacent type dataset, such as the ImageNet [135] database of 3.2 million images. This pre-trained model is then transferred and refitted with your smaller dataset. During this second step, the early hidden node layers of the network are ‘frozen,’ and only deep layer parameter weights can be iteratively modified. Freezing the weights and values of nodes in the first few layers protects fundamental information learned on the large dataset and only allows for ‘fine tuning’ of later nodes so that your desired outcome can be predicted. Transfer learning is widely utilized for clinical outcome prediction [136,137,138]. To facilitate transfer learning, initiatives exist to train large general base models on broad datasets to be utilized for future downstream tasks [139]. An example of such a foundational model includes Med-BERT [140] which is deep neural network model with pre-trained contextual embeddings specific for predicting disease from electronic health records. While experimental, and seemingly poised for powerful clinical modeling, caution prior to implementation is rightfully being taken to understand limitations of the foundational model which would be inherited by all downstream functions [139]. Dropout [141], or randomly removing nodes temporarily during training iterations, can prevent overfitting and improve model performance [142]. Survival neural networks [143,144] exist and are used for predicting time-dependent and censored clinical outcomes [145].

Recurrent neural networks allow for information stored in a node at a previous time point to connect with nodes at later time points. This historical feedback is the hallmark of a recurrent neural network, which allows for sequence or time-series data to be captured. RNNs are used to time-dependent outcomes such as epileptic siezures [146] and cancer treatment response [147]. Two common types of RNN are long short term memory (LSTM [148]) and gated recurrent units (GRU [149]) RNNs which allow for information to be carried and accessed for longer periods without information loss. Convolutional neural networks uniquely capture spatial information within data, and adjacent inputs must be related for CNN to be useful. CNNs have been utilized to predict malignancies from pulmonary nodules [150]. Overall, deep neural networks demonstrate superior performance on nearly all multimodal and image-based classification tasks, but are on par with other methods in regard to purely tabular inputs [151]. A limitation is their interpretability, as no singular features or direct feature weights are carried forward.

## Data extraction and preprocessing

Each of these emerging methods requires access to reliable, standardized data input (X) that is appropriately captured to model an outcome of interest (y) [152]. To obtain and maintain X, extraction pipelines and preprocessing steps much be carefully attended. Often, this is the most time-consuming step in developing an ML model [153]. To predict our outcome ŷ accurately on new seen data, we will need to ensure that our training data X is generalizable [154] and representative of the population for which we aim to perform clinical outcome predictions.

1. *Data Accession and Extraction.* A convenient method of data storage is a clinical repository. Here data is stored in a data frame or table such that X is already formatted with patients listed as rows, and relevant feature variables for each patient are listed as columns. However, the necessary data will often not be available in this format and must be collated and transformed into the proper input format. The 2010 passage of the Affordable Care Act [155] included a mandate for health care providers to adopt electronic health record (EHR) systems. EHR records require a large amount of storage space, and due to their nature, cannot be recorded as one data table. Instead, data is often decentralized and made available through encrypted linking of data lakes [156] (raw unstructured) or data warehouses [157] (semi-structured or structured) data storage. Figure 5 shows how information is stored by hospital systems and can be collated on request or query. To perform clinical predictions, interfacing with these raw outputs, data lakes, and warehouses is required, and currently, several modalities exist to do so. One popular standard is FHIR [158] or the fast healthcare interoperability resource. This API Python coding tool provides a standard format in which, as a researcher, you can submit a query to FHIR, which provides the correct back-end commands to retrieve a properly formatted output table [158]. These tools become particularly useful when trying to concatenate clinical data with genetic sampling data and other individual lab or other biomarker values that might exist in various datasets. In general, such processes outside of FHIR can be accomplished on multiple platforms through merging datasets [159] with overlapping patient instances or concatenating data instances to an already existing data set. FHIR [160] directed EHR extraction to clinical outcome prediction pipelines [161] are incipient, and examples include predicting opioid use after spine surgery [162], outcomes and superiority of chronic disease treatment methods [163], and others [164]. Data extraction, or accession pipelines [162] far more complex than these, are also being explored and implemented to conduct clinical outcome predictions. To circumnavigate inter-institutional competition, privacy, permission, and remote storage issues, the use of blockchain technology for data accession rather than extraction is an emerging method being pursued [165]. Specifically, ‘swarm learning’ allows for decentralization and confidentiality of data to be maintained, which may increase intra-institution EHR center participation, and the overall sample sizes available for clinical outcome predictions [165].
2. *Data Preprocessing.* Now that the raw data is extracted (or accessed) and combined into a meaningful data set representation, the often tedious and challenging work of data cleaning and preprocessing can take place. Inattention to these steps that can lead to inaccurately performing ML models. We will draw on both well-established statistical data preparation methods, and more recent preprocessing approaches to prepare these data. The first consideration should be the appropriateness of features included [166]. Given the natural variance or ‘noise’ of real-world data, features may spuriously have predictive properties in your testing data that are not reflected in real clinical settings. Therefore, ask yourself if the inclusion of a variable makes sense for your outcome of interest and discard features not expected to provide predictive value. Also, ask yourself, ‘how was this variable recorded?’ ML models can draw inferences from variables that may not be expected [167]. For example, if a relatively innocuous feature such as functional status in the clinical data set is only recorded for very sick individuals, then a model predicting death may be weighting that variable for its presence or absence independent of the score itself. After this first crucial step, a second step would involve feature construction [168]. Are there already established metrics or outputs that need to be constructed from the features at hand? Feature construction can be very beneficial to overall model performance [169], as it forces the model to consider combinations of features during training explicitly. Feature transformation [170] is an additionally critical. It is often advantageous to convert data from one form to another, such as continuous to discrete. For example, ‘age’ is a popular continuous variable that can be helpful to bin into discrete intervals. Outlier removal [171] is often warranted when using real-world data, which may include erroneous results or other unhelpful extreme values. Any method to remove outliers, however, should be standardized [171]. Several methods exist, including removing an outlier with a one-class support vector machine [172], a covariance estimator [173], or an isolation forest [174]. Finally, it may be appropriate to perform feature scaling [175], which could involve applying log transformations or other scales to create features with better value distributions and ranges.
3. *Optimizing row instances.* Generally, increasing your training data strengthens final model performance in predicting outcomes [176]. If you have few training instances, it may be appropriate to increase your data set size through resampling methods or synthetic data generation. Resampling methods [177] include different cross-validation procedures which involve more complex partitioning and reuse of training and testing samples. To increase the sample size, you could also create synthetic data. Many statistical methods exist for generating synthetic data [178], such as SMOTE [179], and all of which serve to produce new synthetic samples that are similar but plausibly different from the original ‘true’ samples. Synthetic data allows the model to improve performance by giving additional samples to iterate over while optimizing feature weights. Adding synthetic data can improve the number of minority class samples in the dataset. It is a common challenge of ML modeling for a model to underweight rare minority classes [180]. To force the model to more reliably predict the minority class, you can up-sample that class through synthetic data generation. Alternatively, if your sample size is sufficient, you may down sample the majority class [181] to better balance your input data, although care should be taken not to exclude relevant subgroups. An important distinction is that while synthetic or resampled can be applied to model training data, it is generally not acceptable to include synthetic or resampled data in testing datasets.

Similar to synthetic data generation, the statistical imputation [182] of missing values is a powerful tool during data preprocessing. You may have nearly complete data, where a variable may not be populated for a few samples. Depending on the data type, you can impute the missing data. Imputing is the idea of using the context clues from the surrounding features and what has been observed elsewhere in the dataset to estimate what the missing value or parameter should be. Imputation is common in biological contexts [182].

4. *Optimizing column features.* Metrics such as information gain [183] either through the GINI index, or the information gain ratio [184] are statistical metrics that can be used to determine how much information from a potential input feature is given to the outcome. Features giving relatively no information gain may be candidates for removal. Several methods can capture the information stored in multiple features but convey them in fewer features. This concept is known as dimensionality reduction [185], and it can be useful when consistent standardized data are inaccessible and features are abundant. Statistical methods, such as principal component analysis [186], and unsupervised ML methods, including t-SNE [187], and UMAP [188], can serve this purpose. Also useful for clustering analysis, these methods can reduce high dimensional data into a lower-dimensional representation, to accomplish the dimension reduction goal. These methods can be described as ‘representation learning’ or identifying a lower-dimensional feature to represent higher dimensional data

A common challenge arises when dealing with categorical variables, such as blood type that lack ordinal relationships. Unaltered input of this feature as integer representations (1, 2, 3, etc.) would falsely convey a natural ordering to these data that is not present biologically. To address this, the statistical method of one hot encoding [189] exists, which converts categorical variable features into individual columns for each of its subcomponents. One hot encoding is useful, but it can lead to expansive datasets. As a consequence of training a neural network on a categorical variable, the network ‘embedding’ representation of the categorical variable holds representational value. These categorical embeddings [190] can be extracted from the trained neural network and used in place of a categorical variable to represent the feature, uniquely capturing unobvious inter-feature relationships accurately [191], improving the end trained ML model's performance [192].

## Evaluation

With our outcome identified, method selected, and input data preprocessed, it is time to train our model. We need to separate the data set X into training and testing data. An 80%: 20% training to testing split ratio of the total data is common [193]. In sectioning data, it is useful to ensure that all output classes are represented and any sub-type demographics of interest in both the testing and training sections. ML development occurs on the 80% training set. Model training should never involve the test set [13]. We want the test set to be an objective example of what new data would look like if given to the model. Cross validation [194] and random sampling with replacement [195] are useful repeated sampling metrics to estimate average model performance, in the case your initial train-test split happened to be unusually favorable or unfavorable toward model generation.

During training, the ML method will iteratively attempt to minimize the loss function. Training will stop after a preset number of training iterations, or when a certain loss function threshold is reached. At this point, your model can be trialed against the test data, and the difference between predictions generated on the test data ŷ and the ground truth *y* can be compared. For classification problems, ML models will output a class probability score.

**Figure 6.**
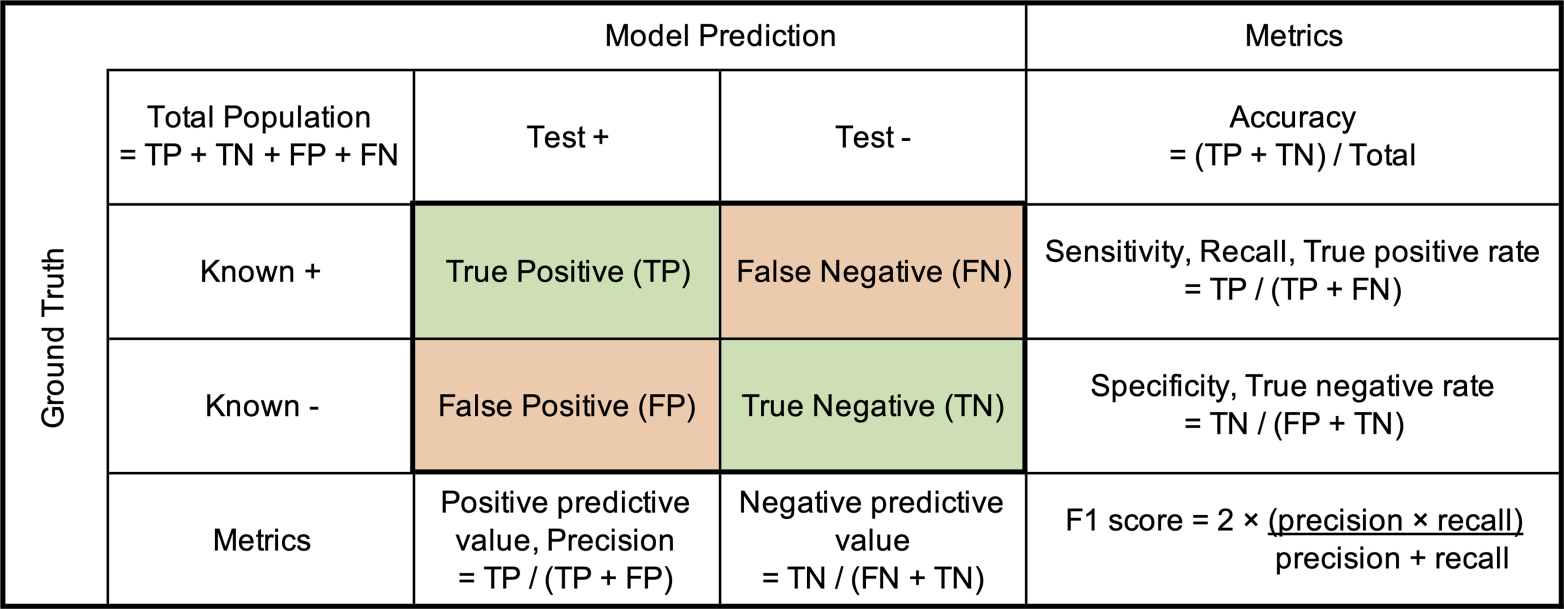
Confusion matrix for model evaluation and formulas for calculating summary statistics.

Many performance evaluation metrics are available for understanding how ŷ compares to *y.* This class probability score can be visualized as an area under the receiver operator curve (AUC –ROC [196,197]) or an area under the precision-recall curve (AUC-PR [198]). Thresholding the probability score will allow for a class prediction to be made. A confusion matrix can next be generated, indicating how many of the test set instances were correctly classified as positive (true positive) or negative (true negative) and incorrectly classified as false positive and false negative outcomes. Figure 6 shows how to calculate relevant outcome summary evaluation metrics from these findings. F1, which is the harmonic mean of precision and recall, is commonly used to compare ML methods, including in cases of class imbalance [199].

## Conclusion and future directions

AI methods are well suited to predict clinical outcomes. A great amount of methodological and application development has occurred and now serves as a precedent, inspiring even further method trialing and advancement. Currently, the methods for cleaning, creating, accessing, extracting, augmenting, and representing data are well defined. Appropriate procedures are available to model different outcome types, and methods are ever evolving to predict clinical outcomes more accurately. We still find that barriers to robust ML implementation into clinical practice, however, remain. The interfaces used for implementing AI methods are undergoing rapid competitive selection. In addition to a coding background and familiarity with statistics, to perform ML well, one needs to have access to professional statistical software (STATA [200], SAS [201], Matlab [202]) or know how to code in R [203] or Python [204]. We view the continued development of user-friendly ML software, including Excel plug ins [203], Orange [205], and KNIME [206] development for visual coding, will increase accessibility, understanding, and use. An additional barrier to robust implementation is regulation. Currently, diagnostic predictors or clinician assist tools are viewed as ‘software-as-medical’ devices [207]. To get regulatory approval, a model must demonstrate superiority in the clinical predictions it creates over a physician or group of physicians. As the FDA navigates these uncharted regulatory waters, perhaps a perspective shift to non-inferiority will occur, allowing for more ML model large-scale adoption in clinical settings. To hire a radiologist, does the new individual need to be better than all the radiologists that came before them? Should this be the same logic used in approving clinician assist and support tools? Finally, ML methods will need to increase in their proof of interpretability, generalizability, and adaptability. As deep learning gets increasingly complex, how can we verify predictions are not being biased? How will models be updated to avoid becoming stagnant, no longer representing shifted populations and parameters? These key questions will need to be addressed for widescale deployment and acceptance of ML clinical outcome predictors going forward. In addition to overcoming these barriers, we suggest the future direction of AI clinical outcome predictions to increase focus on personalized medicine and create strong models that can be used for person-specific goals.

## Summary

- AI methods are well suited to predict clinical outcomes.
- Current methods for cleaning, creating, accessing, extracting, augmenting, and representing data for the use of AI clinical prediction are well defined and ready for implementation.
- The use of AI to predict clinical outcomes is a dynamic and rapidly evolving arena, with new methods and applications emerging.
- Barriers to robust AI clinical outcome prediction include changing AI development interfaces, regulation requirements, and limitations in model interpretability, generalizability, and adaptability over time.

## Abbreviations

AI: Artificial Intelligence
AUC-PR: Area under precision-recall curve
AUC-ROC: Area under receiver operator curve
CNN: Convolutional Neural Network
DL: Deep Learning
EHR: Electronic Health record
FDA: The U.S. Food and Drug Administration
FHIR: Fast Healthcare Interoperability Resources
FN: False Negative
FP: False Positive
GRU: Gated recurrent units recurrent neural network
IoMT: Internet of Medical Things
IoT: Internet of Things
kNN: k-Nearest neighbor
LR: Linear regression
LSTM: Long short term memory recurrent neural network
ML: Machine Learning
NB: Naïve Bayes
PCA: principal component analysis
RNN: Recurrent neural network
SVM: Support vector machines
TN: True Negative
TP: True Positive
t-SNE: t-stochastic neighbor embedding
UMAP: Uniform Manifold Approximation and Projection
