# Supplementary material for: Artificial intelligence, machine learning, and deep learning for clinical outcome prediction
Source: Emerg Top Life Sci. 2021 Dec 20;5(6):729–45. doi: 10.1042/ETLS20210246 (PMC8786279; doi:10.1042/ETLS20210246)
Supplement: Supplementary Table S1 [file ETLS-5-729-s1.pdf]

Supplementary Table 1. Reviews per medical specialty of the current use of machine learning methods in clinical outcome prediction.

Medical Specialty	Review Articles
Anesthesiology[32–34]	Chae et al. 2020, Alexander et al. 2020, Hashimoto et al. 2020
Dermatology[35–37]	Du et al. 2020, Thomsen et al. 2019, Chan et al. 2020
Emergency Medicine[38,39]	Stewart et al. 2021, Tang et al. 2021
Family Medicine[40,41]	Kueper et al. 2020, Ben-Israel et al. 2020
Internal Medicine[41–43]	Ben-Israel et al. 2020, Pandit et al. 2020, Stafford et al. 2020
Interventional Radiology[44,45]	Mazaheri et al 2021, Desai et al. 2021
Medical Genetics[46]	Rauschert et al 2020
Neurological Surgery[47]	Buchlak et al. 2019
Neurology[48-50]	Myszczynska et al. 2020, Sirsat et al. 2020, Yuan et al. 2021
Obstetrics and Gynecology[51,52]	Iftikhar et al. 2020, Sone et al. 2021
Ophthalmology[53-55]	Sengupta et al. 2020, Sarhan et al. 2020, Armstrong et al. 2020
Orthopaedic Surgery[56]	Orink et al. 2021
Otorhinolaryngology[57,58]	Standiford et al. 2021, Crowson et al. 2020
Pathology[59–61]	Thakur et al. 2020, Sultan et al. 2020, McAlpine et al. 2021
Pediatrics[62]	Hoodbhoy et al. 2021
Physical Medicine and Rehabilitation[63,64]	Khera et al. 2020, Amorim et al. 2021
Plastic and Reconstructive Surgery[65,66]	Mantelakis et al. 2021, Huang et al. 2021
Psychiatry[67,68]	Le Glaz et al. 2021, Bracher-Smith et al. 2020
Radiation Oncology[69,70]	Field et al. 2021, El Naqa et al. 2021
Radiology[71,72]	Rajkumar et al. 2020, Wichmann et al. 2020
General Surgery[73,74]	Elfanagely et al. 2021, Henn et al. 2021
Cardiothoracic Surgery[75,76]	Kilic et al. 2020, Dias et al. 2020
Urology[77,78]	Suarez-Ibarrola et al. 2019, Salem et al. 20201
Vascular Surgery[79,80]	Zarkowsky et al. 2021, Boyd et al. 2021
